# Quantitative neurobiological evidence for accelerated brain aging in alcohol dependence

**DOI:** 10.1038/s41398-017-0037-y

**Published:** 2017-12-11

**Authors:** Matthias Guggenmos, Katharina Schmack, Maria Sekutowicz, Maria Garbusow, Miriam Sebold, Christian Sommer, Michael N. Smolka, Hans-Ulrich Wittchen, Ulrich S. Zimmermann, Andreas Heinz, Philipp Sterzer

**Affiliations:** 1 Department of Psychiatry and Psychotherapy, Charité Universitätsmedizin, Berlin, 10117 Germany; 2 Department of Psychiatry and Psychotherapy, Technische Universität Dresden, Dresden, 01069 Germany; 3 Neuroimaging Center, Technische Universität Dresden, Dresden, 01069 Germany; 4 Institute of Clinical Psychology and Psychotherapy, Technische Universität Dresden, Dresden, 01069 Germany

## Abstract

The premature aging hypothesis of alcohol dependence proposes that the neurobiological and behavioural deficits in individuals with alcohol dependence are analogous to those of chronological aging. However, to date no systematic neurobiological evidence for this hypothesis has been provided. To test the hypothesis, 119 alcohol-dependent subjects and 97 age- and gender-matched healthy control subjects underwent structural MRI. Whole-brain grey matter volume maps were computed from structural MRI scans using voxel-based morphometry and parcelled into a comprehensive set of anatomical brain regions. Regional grey matter volume averages served as the basis for cross-regional similarity analyses and a brain age model. We found a striking correspondence between regional patterns of alcohol- and age-related grey matter loss across 110 brain regions. The brain age model revealed that the brain age of age-matched AD subjects was increased by up to 11.7 years. Interestingly, while no brain aging was detected in the youngest AD subjects (20–30 years), we found that alcohol-related brain aging systematically increased in the following age decades controlling for lifetime alcohol consumption and general health status. Together, these results provide strong evidence for an accelerated aging model of AD and indicate an elevated risk of alcohol-related brain aging in elderly individuals.

## Introduction

The premature aging hypothesis posits that alcohol dependence (AD) accelerates aging and that the brains of individuals with AD resemble those of chronologically older healthy individuals^1^. The first neuroanatomical report about a parallel between chronological aging and AD was based on post mortem analyses: Courville^2^ noticed that the cerebral atrophy in brains of individuals with AD resembled the brain shrinkage that occurs with chronological aging. More recent studies have used magnetic resonance imaging (MRI) in individuals with AD and found cortical and subcortical grey matter loss (GML) throughout the brain as compared to healthy controls^3–6^. Here too, qualitative reports have noted that those areas that are particularly susceptible to GML in individuals with AD (in particular frontal regions) overlap with those found for chronological aging^7^. However, to date no study has systematically and quantitatively investigated the similarity of age-related and alcohol-related GML or quantified the extent of brain aging in AD.

To this aim, we developed and applied a novel whole-brain pattern-based approach to analyse grey matter volume information measured with MRI. We used data from a recent study in Germany, in which structural MRI scans were obtained from recently detoxified, abstinent individuals diagnosed with AD (*N* = 119) and a healthy control group (*N* = 97) (see Table 1 for sample characteristics). A parcellation scheme of cortical and subcortical brain regions^8^ served to compare cross-regional GML patterns of AD with patterns of chronological aging. In addition, these patterns served as the basis for a brain aging model of AD.

**Table 1:** Sample characteristics for alcohol-dependent and healthy control subjects

	AD group (*N*=119)			Control group (*N*=97)					
	Mean	SD	%	Mean	SD	%	*t* or *χ* ^2^	df	*p*
Gender (female)			15.1			16.5	0.001	*N*=216	0.98
Age in years	45.0	10.7		43.7	10.8		0.9	214	0.38
SES	−0.4	1.9		0.7	2.1		−3.6	170	<0.001
Lifetime alcohol intake in kg (pure alcohol)	1805	1121		285	810		11.1	214	<0.001
Alcohol intake per drink year in kg (pure alcohol)	55.5	25.8		10.0	23.3		13.4	214	<0.001
Age of AD onset in years (DSM-IV)	32.0	12.0						*N*=111	
Duration of AD in years (DSM-IV)	11.7	9.9						*N*=110	
Abstinence before MRI in days	22.8	11.5						*N*=115	
ADS	14.8	6.9		2.0	3.0		17.0	213	<0.001
OCDS-G total score	11.9	8.5		2.8	2.8		10.1	207	<0.001
Smokers			76.5			67.0	1.9	*N*=216	0.16
FTND (sum score)	3.6	2.8		1.4	2.0		6.4	214	<0.001
WHODAS-II	19.9	6.8		13.5	8.4		8.4	204	<0.001
BIS-15 total score	31.6	6.5		29.1	5.5		2.9	205	0.004
TMT (percentile)	36.1	25.1		44.8	25.1		2.5	209	0.014
DSST	64.3	15.1		73.5	16.6		4.2	211	<0.001
DSB	6.5	1.9		7.4	2.0		3.4	214	0.001
MWT	104.7	9.4		104.5	8.9		−0.2	209	0.82

Socioeconomic status (SES): sum of *z*-transformed self-ratings of social status, household income and inverse personal debt scores^29^; Alcohol Dependence Scale (ADS): degree/level of AD^30^; Obsessive Compulsive Drinking Scale (OCDS-G): current craving for alcohol^31^; Fagerström test for nicotine dependence (FTND): intensity of physical addiction to nicotine; Disability Assessment Schedule 2.0 of the World Health Organization (WHODAS-II): generic assessment instrument for health and disability; Barratt Impulsiveness scale (BIS-15): impulsivity^32^; trail making test (TMT): visual attention and task switching; digit symbol substitution test (DSST): processing speed; digit span backwards (DSB): working memory; multiple-choice vocabulary intelligence test (Mehrfachwahl-Wortschatz-Intelligenztest, MWT): crystallized/verbal intelligence

The premature aging hypothesis has been outlined in two different versions^9^. According to the accelerated aging hypothesis^10^, adverse effects of AD manifest relatively independent of chronological age and are thus to be found across all ages. By contrast, the increased vulnerability hypothesis^11, 12^ places the timing of AD-related neurodegenerative effects and behavioural impairments later in life (mid-40s and older^11^). In addition to our general hypothesis of alcohol-related brain aging, we therefore aimed to answer the following questions arising from the above versions of the premature aging hypothesis: is the extent of putative brain aging stable across the lifespan, as predicted by the accelerated aging hypothesis? Or is the onset of brain aging relatively late in life, as predicted by the vulnerability hypothesis?

## Subjects and methods

### Participants

This study was conducted as part of the LeAD study, a bicentric (Berlin, Dresden) German program investigating the neurobiological basis of AD (www.lead-studie.de; clinical trial number: NCT01679145^13–15^). Pooled across the Berlin and Dresden sites, we assessed 119 individuals aged 20–65 (18 females) meeting criteria of AD according to ICD-10 and DSM-IV-TR and 97 healthy controls aged 21–65 (16 females) matched in terms of age, gender and education (highest school-leaving qualification).

We used the computer-assisted interview version Composite International Diagnostic Interview (CAPI-CIDI^16, 17^) to verify diagnosis criteria of AD in the patient group. For inclusion, individuals with AD had to meet criteria for AD for at least 3 years and had to undergo an inpatient detoxification phase (average duration, ±SEM: 22.8 ± 1 days). Alcohol lifetime consumption (LC) was quantified by the standard drink section of the CAPI-CIDI. The instrument was also used to exclude the possibility of AD in healthy controls.

Exclusion criteria for all subjects were left-handedness (Edinburgh handedness index below 50^18^), contraindications for MRI, and a history of or current neurological or mental disorders (excluding nicotine dependence in both groups and alcohol abuse in individuals with AD, but including abuse of other drugs). Mental disorders were assessed according to DSM-IV axis one as verified by the computer-assisted interview version Composite International Diagnostic Interview, CAPI-CIDI^16, 17^. It was ensured that all subjects were free of psychotropic medication (including detoxification treatment) known to interact with the central nervous system for at least four half-lives. Current non-tobacco/non-alcohol drug abuse was confirmed by means of a dedicated urine test.

On a neuropsychological level, we assessed crystallized intelligence using a standardized vocabulary test in German (Mehrfachwahl‑Wortschatztest-Intelligenztest^19^) and three facets of fluid intelligence: (i) working memory capacity by assessing the digit span backwards task (Digit Span^20^), (ii) executive functioning using the trail making test, TMT A and B^21^, and (iii) processing speed by the digit symbol substitution task (DSST, from the Wechsler Adult Intelligence Scale^20^).

The study was conducted in accordance with the declaration of Helsinki and approved by local ethics committees of the Technische Universität Dresden and the Charité Universitätsmedizin Berlin. All participants provided written informed consent after receiving a complete description of the study.

### MRI acquisition

High-resolution T1-weighted structural MRI scans were acquired on a 3-Tesla Siemens Trio scanner using a magnetization-prepared rapid gradient echo sequence (repetition time: 1900 ms; echo time: 5.25 ms; flip angle: 9°; field of view: 256 × 256 mm^2^; 192 sagittal slices; voxel size: 1 mm isotropic).

### Data analysis

#### Voxel-based morphometry

Data were preprocessed and analysed using SPM12 (http://www.fil.ion.ucl.ac.uk/spm) and VBM 8 (http://dbm.neuro.uni-jena.de/vbm). Images were spatially normalized to a Montreal Neurological Institut (MNI) template, segmented (grey matter, white matter, cerebrospinal fluid) and resampled to 1.5 mm isotropic. To create volumetric grey matter partitions corrected for brain size, normalized grey matter images were modulated through a nonlinear-only transformation, resulting in relative grey matter volume maps^22^. This procedure allowed for analysing the relative differences in regional grey matter volume (ie, corrected for individual brain size). Modulated images were smoothed with an 8 mm isotropic Gaussian kernel.

#### Whole-brain univariate analysis

Three different second-level general linear models were computed to estimate whole-brain grey matter volume effects of (i) AD vs. control, (ii) aging and (iii) alcohol LC. Group differences were assessed by subjecting individual grey matter volume images to a second-level random-effects analysis with the factor group (AD, control), controlling for age, gender, site (Berlin, Dresden), smoking (FTND sum score) and general health status (WHODAS-II). Note that there were no significant whole-brain differences between the sites Berlin and Dresden. The effects of aging on grey matter volume was investigated in the healthy control group by regressing on age, while controlling for gender, site, smoking, general health status and mean yearly intake (kilogram pure alcohol) ingested since the first alcoholic drink. Finally, the relationship between grey matter volume and LC in the AD group was investigated with a regression analysis on LC, while controlling for age, gender, site, smoking and general health status.

#### Atlas-based parcellation and cross-regional correlation analysis

Contrast images of the whole-brain univariate analysis provided the basis for a cross-regional correlation analysis. In a first step, the brain was parcelled into 110 GM areas on the basis of an anatomical atlas (JHU atlas^8^), which included a comprehensive set of both cortical and subcortical brain areas. Next, within each brain region, average grey matter contrast estimates were computed for the three group-level models (group, age, LC): (i) controls > AD; (ii) age < 0 (ie, less grey matter volume with increasing age); (iii) LC < 0. Finally, to assess the cross-regional correspondence between AD diagnosis, age and LC, contrast estimates between these factors were correlated across regions.

#### Brain age model

The goal of the brain age model was to compute the biological brain age of participants on the basis of whole-brain grey matter volume patterns. Regional averages were extracted for the brain regions of the JHU atlas from the original grey matter volume map of each participant. To build the model, in each analysis a multilinear ridge regression (λ = 1.0) with age as the dependent variable was trained on data of the control group. The regression model comprised 110 regressors based on the regional grey matter volume patterns and three regressors for gender, site and smoking (FTND sum score). For control subjects, brain age was predicted in a leave-one-sample-out procedure. To predict brain ages of AD subjects, the model was trained once on the entire set of control subjects.

## Results

### Congruent patterns of age- and AD-related grey matter loss

**Fig. 1:**
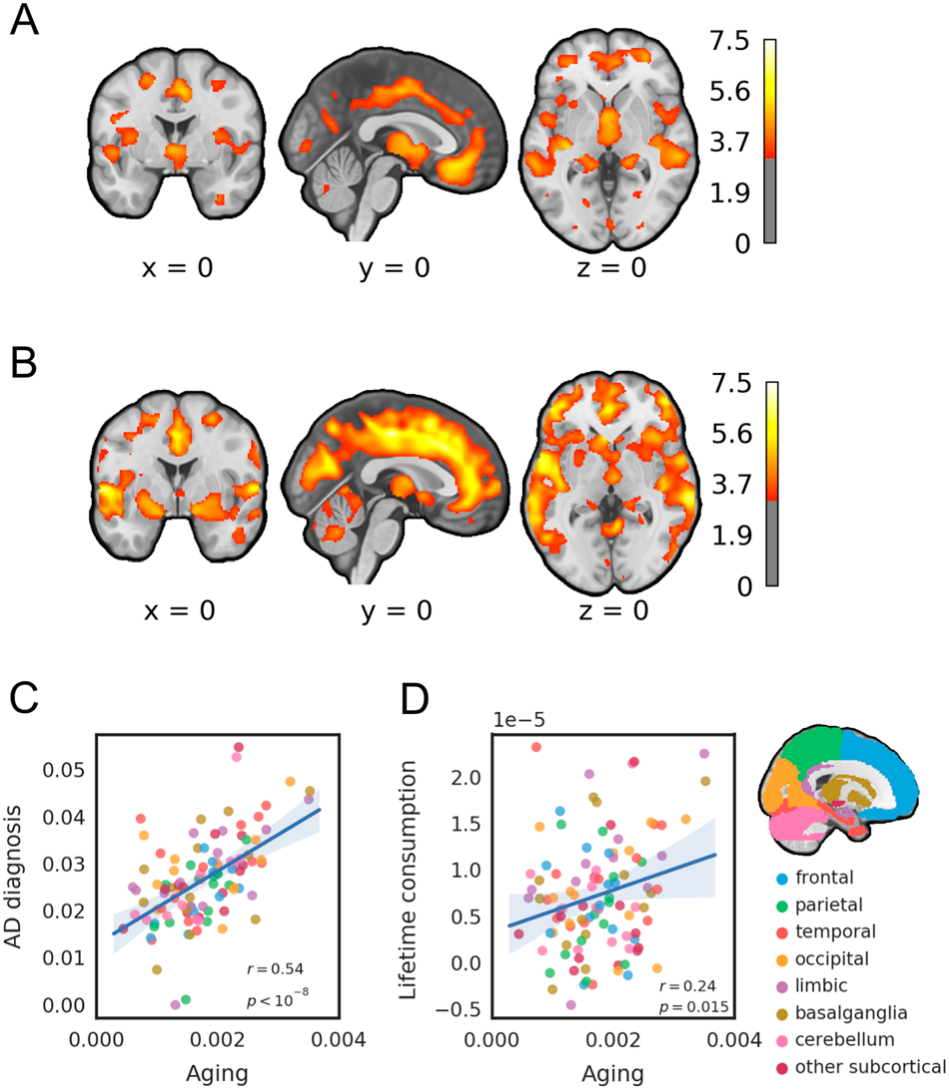
Correspondence between AD-related and age-related grey matter loss (GML). **a** and **b** show t-maps for univariate whole-brain analyses, thresholded at *p* < 0.001 uncorrected, for illustration. **a** T-map for AD-related grey matter volume loss, based on a two-sample *t* test between AD subjects and control subjects, controlling for age, gender, site, smoking (FTND sum score) and general health status (WHODAS-II). **b** T-map for age-related grey matter volume loss in control subjects using a regression analysis controlling for gender, site, smoking, general health status and mean yearly intake (kilogram pure alcohol) ingested since the first alcoholic drink. **c** and **d** show the cross-regional similarity between AD-related and age-related GML. Each data point corresponds to one of 110 anatomical brain regions. Colours indicate regions pertaining to different parts of the brain, as indicated by the map on the right. Age-related GML was derived from the contrast estimates in **b**. **c** Cross-regional relationship between age-related GML and GML associated with the group contrast control > AD of **a**. **d** Cross-regional relationship between age- and consumption-related GML (lifetime consumption). Consumption-related GML was computed as the contrast estimate of a negative relationship between grey matter volume and kilogram lifetime consumption, controlling for age, gender, site, smoking and general health status

In a first step, we computed whole-brain statistical maps for group- and age-related GML based on regional grey matter volume. Fig. 1a, b and Supplementary Tables 1 and 2 show that both AD group membership and chronological aging were associated with widespread and qualitatively similar patterns of GML across the brain, strongly affecting frontal (especially cingulate cortex and middle frontal gyrus), superior temporal and cerebellar areas. To quantitatively assess the cross-regional similarity between age- and AD-related GML, we extracted the average contrast estimates for both effects within each region of the JHU brain atlas^8^ and correlated them across regions. This approach revealed a strong linear relationship between age-related and AD-related GML across 110 anatomical brain regions (*r*_Pearson_ = 0.54, *p*_214_ < 10^−8^) (Fig. 1c).

A possible concern is that the correlation between age- and AD-related grey matter loss might be inflated by the fact that the magnitude of grey matter loss in different regions primarily depends on the size or the general variance of the region. To account for these possibilities, we approximated region size by counting the number of grey matter voxels in each region. Across-subject variance of grey matter volume was computed within the control group for each region individually. A partial correlation approach showed that the correlation also held when controlling for region size (*r*_Pearson_ = 0.56, *p*_214_ < 10^−9^), variance (*r*_Pearson_ = 0.36, *p*_214_ < 10^−4^) or both (*r*_Pearson_ = 0.34, *p*_214_ < 10^−3^). Thus, aging and AD similarly affected regional GML across a comprehensive set of 110 anatomical brain regions even when controlling for region size and interindividual variance.

In a next step, we investigated whether individual LC within the AD group would likewise be reflected in an age-like cross-regional pattern. Using contrast estimates for a negative linear relationship between LC and grey matter volume, we found a clear correspondence between GML patterns of LC and age (*r*_Pearson_ = 0.24, *p*_214_ = 0.015) (Fig. 1d). Thus, age-related GML patterns were similar to alcohol-related GML patterns both in terms of a between-group diagnostic contrast and a within-group consumption-based contrast.

### Increased brain age in AD subjects

**Fig. 2:**
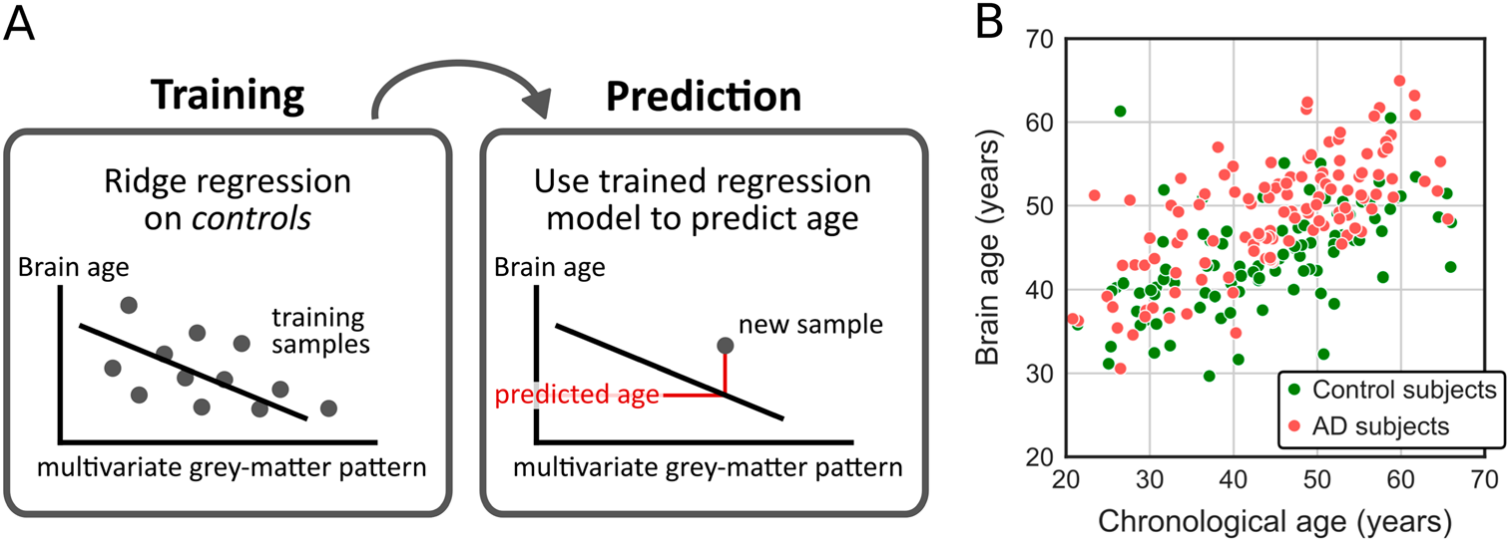
Brain age. **a** Brain age model. A ridge regression model was trained on the grey matter patterns of control subjects and served to predict the brain age of AD subjects. **b** Chronological age vs. predicted brain age in AD and control subjects

While the above results hint at an accelerated aging process in brains of AD subjects, they leave open the extent of such an acceleration; in other words, by how much does the brain age of AD subjects increase? To answer this question, we trained a multilinear ridge regression model on the grey matter volume patterns of the control group with chronological age as the dependent variable (Fig. 2a). For an initial verification, we first tested the age model within the control group. A leave-one-out cross-validation procedure was used, such that in each of N folds the model was trained on N−1 control subjects and predicted the age of the left-out control subject. We found that the predicted age was strongly related to the chronological age (*r*_Pearson_ = 0.54, *p* < 10^−7^; average predicted age: mean ± SEM = 43.7 ± 1.1 years; average chronological age: 43.7 ± 0.6; mean absolute error: 6.9 years) (Fig. 2b), thus affirming the general validity of the model.

We then trained the brain age model on all control subjects and applied it to AD subjects. We found that the brain age of AD subjects was increased by 4.0 ± 0.7 years relative to their chronological age (predicted age: 49.0 ± 0.6; chronological age: 45.0 ± 1.0; mean absolute error: 6.7 years). This increase was significant (one-sample *t* test: *t*_118_ = 5.6, *p* < 10^−6^). In an exploratory analysis, we investigated brain aging in AD subjects for different regions of the brain, which revealed that limbic, temporal and frontal were numerically most strongly affected (Fig. S1). To ensure that the model was generally suited for the AD group, we confirmed that, despite the predicted age gap, the predicted age and the chronological age of AD subjects were strongly correlated (*r*_Pearson_ = 0.69, *p*_214_ < 10^−17^) (Fig. 2b). These results provide clear evidence for accelerated aging in the brains of AD subjects.

### Brain aging increases with lifetime alcohol consumption and age

Finally, we assessed to which degree brain aging (predicted age minus chronological age) in AD subjects was affected by the amount of LC and chronological age.

First, we regressed brain aging on LC, accounting for age. We found that 1 kg (or 71 standard drinks of 14 g) of alcohol intake corresponded to approximately half a day of brain aging in AD subjects (*β* = 0.56 ± 0.25, *p*_214_ = 0.028). Thus, the degree of brain aging is predicted by the amount of alcohol consumed throughout life.

**Fig. 3:**
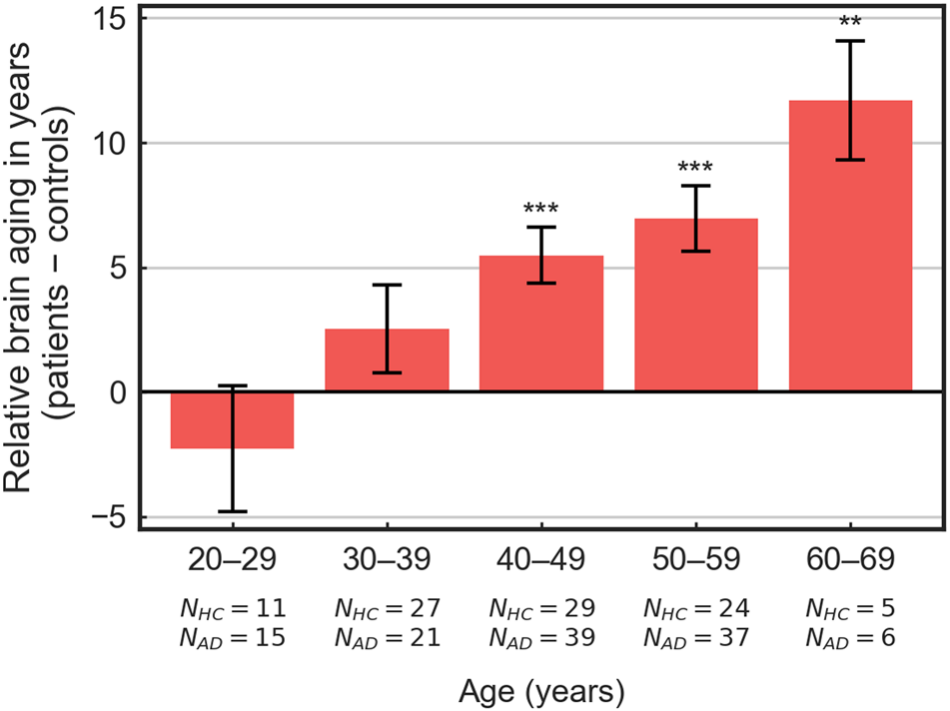
Brain aging in dependence of chronological age. Brain aging of AD subjects in comparison to control subjects for five life decades. Mean values indicate the difference of the group means (AD group minus control group); error bars indicate the pooled standard error of the group differences; **: < 0.01; ***: < 0.001

Second, we assessed the relationship between brain aging in AD and chronological age. Since brain age estimates were biased with respect to chronological age irrespective of group (controls: *r*_Pearson_ = −0.82, *p*_214_ < 10^−24^; AD: *r*_Pearson_ = −0.77, *p*_214_ < 10^−23^; see also Fig. 2b), we compared brain aging in AD subjects directly to age-matched control subjects. After regressing out gender, LC, smoking and general health status (WHODAS-II) from brain aging estimates, we sorted AD and control subjects into five chronological decades and submitted the brain aging estimates to a two-way (2 × 5) analysis of variance with factors group and decade. This analysis revealed main effects of group (*p* < 10^−6^, *F*_1,43_ = 27.8) and age (*p* < 10^−15^, *F*_1,68_ = 60.0) as well as an interaction of group and age (*p* < 0.001, *F*_4,2_ = 5.0) (Fig. 3). A post hoc *t* test for the hypothesis of linearly increasing brain aging in AD subjects but not control subjects (contrast vector: [−2, −1, 0, 1, 2; 0, 0, 0, 0, 0]), was likewise significant (*p* < 10^−6^, *t*_214_ = 5.5). Thus, brain aging in AD subjects increased with chronological age. While brain aging was not significant in the range 20–29, it was estimated as high as 11.7 ± 2.4 years in the ages 60–69.

## Discussion

Our whole-brain analyses revealed that both AD and aging reduced grey matter volume in largely overlapping brain areas, in particular frontal (cingulate cortex and middle frontal gyrus), cerebellar and superior temporal regions. We quantitatively substantiated this parallel by showing a striking correlation between regional alcohol-related and age-related GML patterns. A brain age model built on grey matter patterns showed substantial brain aging in the AD group, which increased with LC and chronological age.

The strong similarity between AD- and age-related GML invites two possible, nonexclusive interpretations. First, it may be that the neurotoxic effects of excessive alcohol intake are, at a fundamental biological level, comparable to deteriorating effects of the aging brain. While the exact pathological molecular mechanisms of alcohol-related neuronal damage have not been revealed yet^23^, prominent candidate mechanisms are processes that alter cell-integrity such as (chronic) oxidative stress^24^. Indeed, oxidative stress has been found to increase both with aging^25^ and (in model organisms) with excessive ethanol exposure^26^. Crucially, if different brain regions vary in their vulnerability to such a common biological mechanism, similar regional patterns of age-related and AD-related GML as observed in the present study are the consequence.

Second, different brain areas might be generally more or less susceptible to grey matter loss irrespective of a specific neurodegenerative mechanism. In this case, one may expect to find similar cross-regional profiles across a variety of illnesses that affect grey matter. A potential avenue for future research is thus to investigate whether other factors that cause GML, such as chronic stress or psychiatric and neurodegenerative disorders, exhibit patterns of GML that are likewise comparable to the pattern of the aging brain. A recent study^27^ provides initial evidence for this possibility, by showing aging-like changes in brain structure for a range of psychiatric disorders (schizophrenia, major depression and borderline personality disorder). Such future research would clarify whether the similarity to age-related GML is indeed specific to AD.

The similarity between age- and AD-related GML patterns provided motivation for a brain age model of AD. Our results revealed an average increase of the brain age of 4 years relative to chronological age, thus demonstrating that alcohol-related brain aging was substantial in relation to the human lifespan. Of note, despite its simplicity, the accuracy of the model with respect to the age prediction in control subjects was on a competitive basis with more complex approaches^28^. Our results thus confirm and quantify, for the first time, accelerated alcohol-related brain aging on a chronological scale. Moreover, relating brain aging to LC, we found that each kg of alcohol consumption corresponded to approximately half a day of brain aging. This result provides further validation for the brain aging model and may be particularly useful for psychoeducational purposes.

An analysis of brain aging as a function of chronological age revealed a systematic increase of brain aging over the lifespan. While brain aging was highest in the oldest AD subjects of our cohort (ages 60–69; 11.7 ± 2.4 years), no brain aging was detectable in the youngest AD subjects (ages 20–29). These results resonate with both the vulnerability hypothesis and the accelerated aging hypothesis. In line with the accelerated aging hypothesis (but contrary to the vulnerability hypothesis), brain aging was measurable throughout the lifetime, with the exception of only the youngest AD subjects tested. On the other hand, the results did show more pronounced alcohol-related brain aging with increasing chronological age. This pattern, as well as the indication of protective factors in the youngest AD subjects, are in accordance with the vulnerability hypothesis. Overall, our results thus suggest a middle ground between the accelerated aging hypothesis and the vulnerability hypothesis, evidencing accelerated brain aging in all but the youngest individuals with AD and a progressive vulnerability to brain aging with increasing chronological age.

Limits of the present study are the relatively small number of females (16%) in this study, potentially masking effects of gender, and possible side effects of physical or mental comorbidities on GML, that may have not been fully prevented by controlling for general health status (WHODAS-II) and by excluding participants with non-AD mental disorders.

In conclusion, the present study provides novel neurobiological evidence for accelerated aging in AD, casting the neurotoxic effects of alcohol as an effective increase of brain age. In addition, it demonstrates that over and above total grey matter volume, cross-regional grey matter patterns are a useful marker of AD.

## Electronic supplementary material

Supplementary Table 1

Supplementary Table 2

Supplementary Figure 1
