# Supplementary material for: Quantitative neurobiological evidence for accelerated brain aging in alcohol dependence
Source: Transl Psychiatry. 2017 Dec 11;7:1279. doi: 10.1038/s41398-017-0037-y (PMC5802586; doi:10.1038/s41398-017-0037-y)
Supplement: Supplementary file 1 — Supplementary Table 1 [file 41398_2017_37_MOESM1_ESM.docx]

| **Supplementary Table 1.** Brain regions with higher regional grey-matter in the univariate whole-brain contrast control > AD (threshold: cluster size k > 100, p < 0.05, corrected for family-wise errors at the whole-brain level). 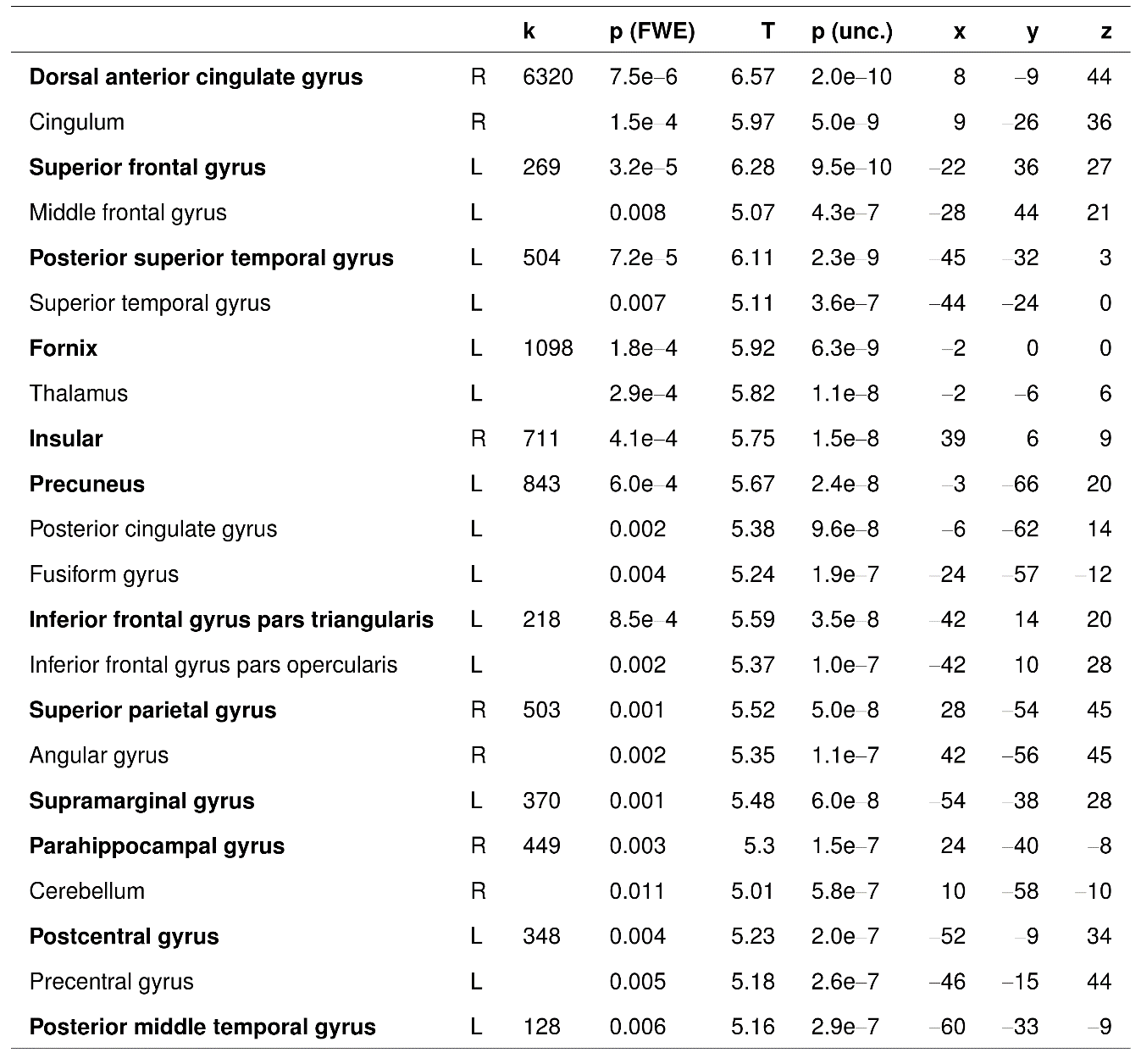 |
| --- |
