# Supplementary material for: Quantitative neurobiological evidence for accelerated brain aging in alcohol dependence
Source: Transl Psychiatry. 2017 Dec 11;7:1279. doi: 10.1038/s41398-017-0037-y (PMC5802586; doi:10.1038/s41398-017-0037-y)
Supplement: Supplementary file 3 — Supplementary Figure 1 [file 41398_2017_37_MOESM3_ESM.docx]

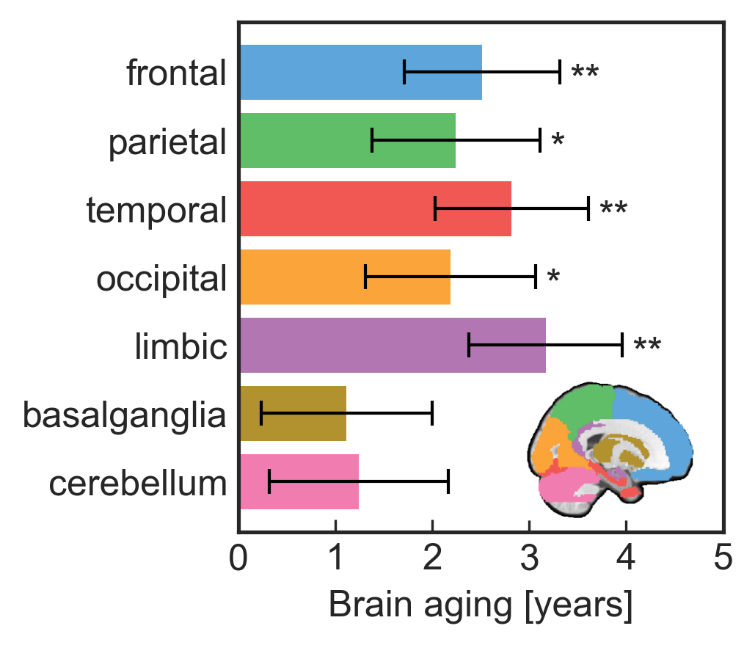


**Supplementary Figure S1.** **Brain aging in patients for different regions of the brain.** Error bars indicate standard error of the mean. Asterisks indicate statistical significance based on a one-sample t-test. * p < 0.05, uncorrected; ** p < 0.05, Bonferroni-corrected for 7 regions.
